# Development of a multiplex RT-PCR method for the detection of four porcine enteric coronaviruses

**DOI:** 10.3389/fvets.2022.1033864

**Published:** 2022-11-08

**Authors:** Jia-Wei Niu, Jin-Hui Li, Jin-Lian Guan, Ke-Hui Deng, Xiu-Wu Wang, Gen Li, Xia Zhou, Min-Sheng Xu, Rui-Ai Chen, Shao-Lun Zhai, Dong-Sheng He

**Affiliations:** ^1^Key Laboratory of Zoonosis Prevention and Control of Guangdong Province, College of Veterinary Medicine of South China Agricultural University, Guangzhou, China; ^2^Ministry of Agriculture of Rural Affairs, Key Laboratory of Animal Disease Prevention of Guangdong Province, Institute of Animal Health, Guangdong Academy of Agricultural Sciences, Scientific Observation and Experiment Station of Veterinary Drugs and Diagnostic Techniques of Guangdong Province, Guangzhou, China; ^3^Zhaoqing Branch Center of Guangdong Laboratory for Lingnan Modern Agricultural Science and Technology, Zhaoqing, China

**Keywords:** porcine epidemic diarrhea virus, porcine deltacoronavirus, porcine transmissible gastroenteritis virus, swine acute diarrhea coronavirus, multiplex RT-PCR

## Abstract

Porcine enteric coronaviruses are pathogens that cause viral diarrhea in pigs and are widely prevalent worldwide. Moreover, studies have shown that some porcine enteric coronaviruses can infect humans and poultry. In order to effectively monitor these viruses, it is necessary to establish a multiple detection method to understand their prevalence and conduct in-depth research. Common porcine enteric coronaviruses include Porcine epidemic diarrhea virus (PEDV), Porcine transmissible gastroenteritis virus (TGEV), Porcine delta coronavirus (PDCoV), and Swine acute diarrhea syndrome coronavirus (SADS-CoV). Pigs infected with these viruses have the common clinical symptoms that are difficult to distinguish. A quadruplex RT-PCR (reverse transcription-polymerase chain reaction) method for the simultaneous detection of PEDV, PDCoV, TGEV and SADS-CoV was developed. Four pairs of specific primers were designed for the PEDV *M* gene, PDCoV *N* gene, TGEV *S* gene and SADS-CoV *RdRp* gene. Multiplex RT-PCR results showed that the target fragments of PDCoV, SADS-CoV, PEDV and TGEV could be amplified by this method. and the specific fragments with sizes of 250 bp, 368 bp, 616 bp and 801 bp were amplified, respectively. This method cannot amplify any fragment of nucleic acids of Seneca Valley virus (SVV), Porcine Reproductive and Respiratory Syndrome Virus (PRRSV) and Atypical Porcine Pestivirus (APPV), and has good specificity. The lowest detection limits of PDCoV, PEDV, TGEV and SADS-CoV were 5.66 × 10^5^ copies/μL, 6.48 × 10^5^ copies/μL, 8.54 × 10^5^ copies/μL and 7.79 × 10^6^ copies/μL, respectively. A total of 94 samples were collected from pig farms were analyzed using this method. There were 15 positive samples for PEDV, 3 positive samples for mixed infection of PEDV and PDCoV, 2 positive samples for mixed infection of PEDV and TGEV, and 1 positive sample for mixed infection of PEDV, TGEV, and PDCoV. Multiplex RT-PCR method could detect four intestinal coronaviruses (PEDV, PDCoV, TGEV, and SADS-CoV) in pigs efficiently, cheaply and accurately, which can be used for clinical large-scale epidemiological investigation and diagnosis.

## Introduction

Viral diarrhea seriously endangers the pig industry throughout the world, and has been one of the problems that has plagued the breeding industry all over the world. It is characterized by acute diarrhea, vomiting, dehydration and high mortality in neonatal piglets, resulting in enormous economic losses (1–3). The pathogens associated with viral diarrhea disease in piglets are mainly coronaviruses, including TGEV, PEDV, PDCoV, and SADS-CoV (4–9). These swine enteric viruses cause similar clinical symptoms in infected pigs, leading to difficulties in diagnosing diarrhea (10).

PEDV and TGEV are two traditional diarrhea pathogens (11). PEDV and TGEV are both unsegmented single-stranded positive-stranded RNA viruses, and both belong to the order *Nidovirales*, the family *Coronaviridae*, and the genus *alpha-coronavirus* (12). Both of two viruses can cause severe diarrheal disease in affected pigs, and the clinical symptoms are mainly acute and severe watery diarrhea, vomiting and dehydration, but the effect of PEDV on 3–4 weeks old piglets is more obvious. In addition, in farms with poor conditions, PEDV and TGEV usually show a trend of mixed infection, and there is the possibility of co-morbidity.

PDCoV and SADS-CoV are two newly discovered coronaviruses in recent years. PDCoV and SADS-CoV are also unsegmented single-stranded positive-stranded RNA viruses. PDCoV belongs to the order *Nidovirales*, the family *Coronavirida*e, and the genus *delta-coronavirus* (13); SADS-CoV belongs to the order *Nidovirales*, the family *Coronaviridae*, and the genus *alpha-coronavirus*. The clinical symptoms caused by PDCoV and SADS-CoV are similar to those caused by other known porcine enteric coronaviruses (12, 14). In 2012, PDCoV was first reported in Hong Kong. It was detected in the feces of diarrhea piglets and sows in the United States in February 2014. Subsequently, the virus was found in the United States, Canada, South Korea, India and Thailand, showing a trend of widespread global spread (15). More notably, research data since 2017 have revealed cross-species transmission and potential zoonotic diseases of swine δ coronavirus from pigs to humans (16). In 2018, SADS-CoV was first reported in Guangdong, China. At present, a large number of piglets have died, and the virus has also been detected in bats in other parts of Guangdong (14).

There are several serological detecting methods currently available for the detection of viruses, such as the immunofluorescence technique, immunochromatography and indirect immunofluorescence assays, but these techniques are time-consuming and unsuitable for testing large-scale samples. Currently, polymerase chain reaction (PCR), real-time PCR, loop-mediated isothermal amplification (LAMP), and enzyme-linked immunosorbent assay (ELISA) methods have been reported for the detection of these viruses (17), these viruses are highly pathogenic in piglets with immature immune systems and few antibodies, so ELISA is less efficient at detecting these viruses than PCR. However, none of the existing RT-PCR methods can simultaneously distinguish between these four viruses. Therefore, in order to diagnose the pathogens quickly and effectively, it is particularly important to establish a rapid and sensitive detection method for the four viruses (18–20). The multiplex RT-PCR method is to detect and identify multiple pathogens at the same time through one RT-PCR reaction (21, 22). The advantages of this method are that it has a wide range of usage environments, excellent specificity and low price. It is more suitable for rapid diagnosis of mixed infections in epidemics, and provides a rapid and accurate diagnostic method for epidemiological investigations and veterinary clinical diagnosis.

## Materials and methods

### Construction of plasmid standards

Before establishing the multiplex RT-PCR assay, the single-plex RT-PCR method for each virus was established using the cDNA of each virus as a template. According the following program: 95 °C for 5 min; 95 °C for 30 s, 55 °C for 30 s, 72 °C 1 min for 30 cycles; 72 °C for 10 min. Multiplex RT-PCR consists of the following components: 10 × Buffer, dNTPs (2.5 mM), TaKaRa Taq (5 U/μL), RNAse-free ddH2O, primers and cDNA. Then, these amplified target fragments of were then individually cloned into the pMD19-T vector. Sequencing confirmed that the recombinant plasmids pMD-19-T-PEDV, pMD-19-T-TGEV, pMD-19-T-SADS-CoV and pMD-19-T-PDCoV contained the target fragments.

### RNA extraction and reverse transcription

Samples positive for TGEV, PEDV, PDCoV, and SADS-CoV were stored in our laboratory. Clinical samples collected from were stored at −80°C. Then, samples were mixed with supernatant by vortexing and collected after centrifugation at 12,000 × g at 4 °C for 15 min. Viral nucleic acids were extracted using the Viral DNA/RNA Kit (Hangzhou Bioer Technology Co. Ltd), and the Reverse Transcriptase M-MLV (RNase H-) was used to perform reverse transcription following the manufacturer's instructions.

### Primer sequences

TGEV, PEDV, PDCoV and SADS-CoV sequences available in GenBank were analyzed to improve the detection performance of primers. Finally, we designed primers with PEDV *M* gene, PDCoV *N* gene, TGEV *S* gene and SADS-CoV *RdRp* gene as conserved genes. Specific primers for the construction of plasmid standards were designed using primer 5 (Version 5.00) (Table 1).

**Table 1:** Primer sequences for TGEV, PEDV, PDCoV, and SADS-CoV.

**Primer**	**Sequence (5^′^-3^′^)**	**Gene**	**Product size**
TGEV–F	GTATGAAGCGTAGTGGTTATGGTC	S	801 bp
TGEV–R	AATAGGTTATGACAGGTTCACAATC		
PEDV–F	TTTCACATGGAATATCATACTGAC	M	616 bp
PEDV–R	ATGAAGCACTTTCTCACTATCTGT		
SADS–F	TCCTGAGGAAGAGGTTGAGATGG	RdRp	368 bp
SADS–R	CGTGCTTACCATTGTGTATGAGAC		
PDCoV–F	AGACACTGAGAAGACGGGTATGG	N	250 bp
PDCoV–R	CTTCTTGTCCTTAGTTGGTTTGGT		

### Reaction condition optimization for multiplex RT-PCR

The optimization was performed on a Biometra TOne 96G PCR instrument based on the following program: 95 °C 5 min; 95 °C 30 s, 55 °C 30 s, 72 °C 1 min 30 cycles; 72 °C 10 min. Multiplex RT-PCR consists of the following components: 10 × Buffer, dNTPs (2.5 mM), TaKaRa Taq (5 U/μL), RNAse-free ddH_2_O, primers and positive plasmids for TGEV, PEDV, PDCoV and SADS-CoV. To obtain the best amplification efficiency, the multiple reaction system was optimized by using different concentrations of primers, dNTPs (2.5 mM) and TaKaRa Taq (5 U/μL), and different annealing temperatures.

### Sensitivity of the multiplex RT-PCR assay

To analyze the sensitivity of established multiplex RT-PCR, standard plasmids for TGEV, PEDV, SADS-CoV, and PDCoV prepared above were mixed. Then, the mix was diluted by 10 gradients with RNAse-free ddH_2_O. The initial concentrations of the four standard plasmids were 8.54 × 10^9^ copies/μL, 6.48 × 10^9^ copies/μL, 7.79 × 10^9^ copies/μL, and 5.66 × 10^9^ copies/μL, respectively. The susceptibility of multiplex RT-PCR to the four viruses was assessed using the diluted plasmids as templates.

### Specificity of the quadruplex RT-PCR assay

The specificity of multiplex RT-PCR was assessed. RNA was extracted from positive samples of PRRSV, Atypical swine fever virus (APPV), Seneca valley virus (SVV), TGEV, PEDV, PDCoV and SADS-CoV preserved in our laboratory, and the RNA was reverse transcribed into cDNA. Multiplex RT-PCR amplifications were performed using cDNA from these viruses and RNAse-free water as templates.

### Reproducibility test of the multiplex RT-PCR assay

In this experiment, 10^6^ copies/μL recombinant plasmid standard was selected and mixed in equal proportions. The stability and repeatability of the quadruple RT-PCR method were verified by seven repeated tests.

### Detection in clinical samples

Ninety four clinical samples (Table 2) collected from pig farms in Guangdong province from 2021 to 2022 were detected. All samples were diluted three-fold with phosphate buffered saline (PBS) using a vortexer and incubated at 4000 ×g for 15 min at 4°C. Total RNA from clinical samples was extracted by the above method. The supernatant was collected and used for RNA extraction and prepared as cDNA using reverse transcription. Then, all cDNA were detected using multiplex RT-PCR of this study. The results of clinical samples detected by the quadruple RT-PCR were repeatedly verified by conventional single RT-PCR to compare the coincidence rate of the two detection methods.

**Table 2:** Types of clinical samples and test results.

**Sample**	**One pathogen positive sample**				**Two pathogens positive sample**		**Three pathogens positive sample**
	**PEDV**	**PDCoV**	**TGEV**	**SADS-CoV**	**PDCoV/PEDV**	**TGEV/PEDV**	**PDCoV/PEDV/TGEV**
FEC1	-	-	-	-	-	-	-
FEC2	-	-	-	-	-	-	-
FEC3	-	-	-	-	-	-	-
FEC4	-	-	-	-	-	-	-
FEC5	-	-	-	-	-	-	-
FEC6	-	-	-	-	-	-	-
FEC7	-	-	-	-	-	-	-
FEC8	-	-	-	-	-	-	-
FEC9	-	-	-	-	-	-	-
FEC10	-	-	-	-	-	-	-
FEC11	-	**+**	-	-	-	-	-
FEC12	-	-	-	-	-	-	-
FEC13	-	-	-	-	-	-	-
FEC14	-	-	-	-	-	-	-
FEC15	-	-	-	-	-	-	-
FEC16	-	-	-	-	-	-	-
FEC17	-	-	-	-	-	**+**	-
FEC18	-	-	-	-	-	-	-
FEC19	-	-	-	-	-	-	-
FEC20	-	-	-	-	-	-	-
FEC21	-	-	-	-	-	-	-
FEC22	-	-	-	-	-	-	-
FEC23	-	-	-	-	-	-	-
FEC24	-	-	-	-	-	-	-
FEC25	-	-	-	-	-	-	-
FEC26	-	-	-	-	-	-	-
FEC27	-	-	-	-	-	-	-
FEC28	-	-	-	-	-	-	-
FEC29	-	-	-	-	-	-	-
FEC30	-	-	-	-	-	-	-
FEC31	-	-	-	-	-	-	-
FEC32	-	-	-	-	-	-	-
FEC33	-	-	-	-	-	-	**+**
FEC34	**+**	-	-	-	-	-	-
FEC35	-	**+**	-	-	-	-	-
FEC36	-	-	-	-	-	-	-
FEC37	**+**	-	-	-	-	-	-
FEC38	**+**	-	-	-	-	-	-
FEC39	**+**	-	-	-	-	-	-
FEC40	-	**+**	-	-	-	-	-
FEC41	-	-	-	-	-	-	-
FEC42	-	-	-	-	-	-	-
FEC43	-	-	-	-	-	-	-
FEC44	-	-	-	-	-	-	-
FEC45	-	-	-	-	-	-	-
FEC46	-	-	-	-	**+**	-	-
FEC47	-	-	-	-	**+**	-	-
FEC48	-	-	-	-	-	-	-
IS1	-	-	-	-	-	-	-
IS2	-	-	-	-	**+**	-	-
IS3	-	-	-	-	-	-	-
IS4	-	-	-	-	-	-	-
IS5	-	-	-	-	-	-	-
IS6		-	-	-	-	-	-
IS7	**+**	-	-	-	-	-	-
IS8	-	-	-	-	-	-	-
IS9	-	-	-	-	-	-	-
IS10	-	-	-	-	-	-	-
IS11	-	-	-	-	-	-	-
IS12	-	-	-	-	-	-	-
IS13	**+**	-	-	-	-	-	-
IS14	**+**	-	-	-	-	-	-
IS15	-	-	-	-	-	-	-
IS16	**+**	-	-	-	-	-	-
IS17	-	-	-	-	-	-	-
IS18	-	-	-	-	-	-	-
IS19	**+**	-	-	-	-	-	-
IS20	-	-	-	-	-	-	-
IS21	**+**	-	-	-	-	-	-
IS22	**+**	-	-	-	-	-	-
IS23	-	-	-	-	-	**+**	-
IS24	-	-	-	-	-	-	-
IS25	-	-	-	-	-	-	-
IS26	-	-	-	-	-	-	-
IS27	-	-	-	-	-	-	-
IS28	-	-	-	-	-	-	-
IS29	**+**	-	-	-	-	-	-
IS30	-	-	-	-	-	-	-
FEC31	-	-	-	-	-	-	-
FEC32	-	-	-	-	-	-	-
FEC33	-	-	-	-	-	-	-
FEC34	-	-	-	-	-	-	-
FEC35	-	-	-	-	-	-	-
FEC36	-	-	-	-	-	-	-
FEC37	-	-	-	-	-	-	-
FEC38	-	-	-	-	-	-	-
FEC39	-	-	-	-	-	-	-
FEC40	-	-	-	-	-	-	-
FEC41	**+**	-	-	-	-	-	-
FEC42	-	-	-	-	-	-	-
FEC43	-	-	-	-	-	-	-
FEC44	-	-	-	-	-	-	-
FEC45	**+**	-	-	-	-	-	-
FEC46	**+**	-	-	-	-	-	-

Results: “+”: Positive; “–”: Negative.

Sample types: FEC, Feces specimens; IS, intestine specimens.

## Results

### Establishment of monoplex RT-PCR reactions for TGEV, PEDV, SADS-CoV and PDCoV

**Figure 1:**
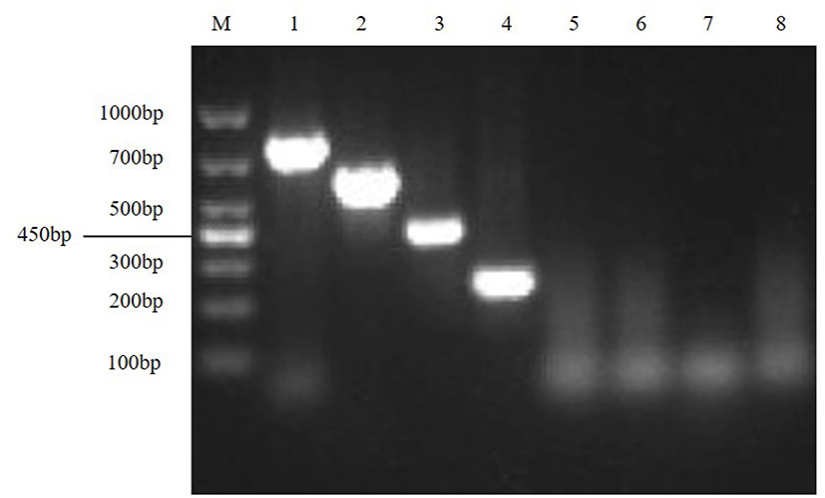
Establishment of monoplex RT-PCR reactions for TGEV, PEDV, SADS-CoV and PDCoV. This figure shows the results of monoplex PCR reactions of TGEV, PEDV, SADS-CoV and PDCoV. Among them, lane M is DNA Marker DL1000, lanes 1–4 represent the monoplex RT-PCR results of TGEV, PEDV, SADS-CoV and PDCoV, respectively; lanes 5–8 represent the negative controls of TGEV, PEDV, SADS-CoV, and PDCoV, respectively.

The results showed that TGEV, PEDV, SADS-CoV and PDCoV showed specific amplification at 801, 616, 368, 263, and 250 bp. The reaction did not produce other miscellaneous bands, indicating that the primer set has good reliability and specificity. And the accuracy of the amplified product was further confirmed by sequencing analysis (Figure 1).

### Optimization of the multiplex reaction system

#### Optimize multiplex RT-PCR annealing temperature

**Figure 2:**
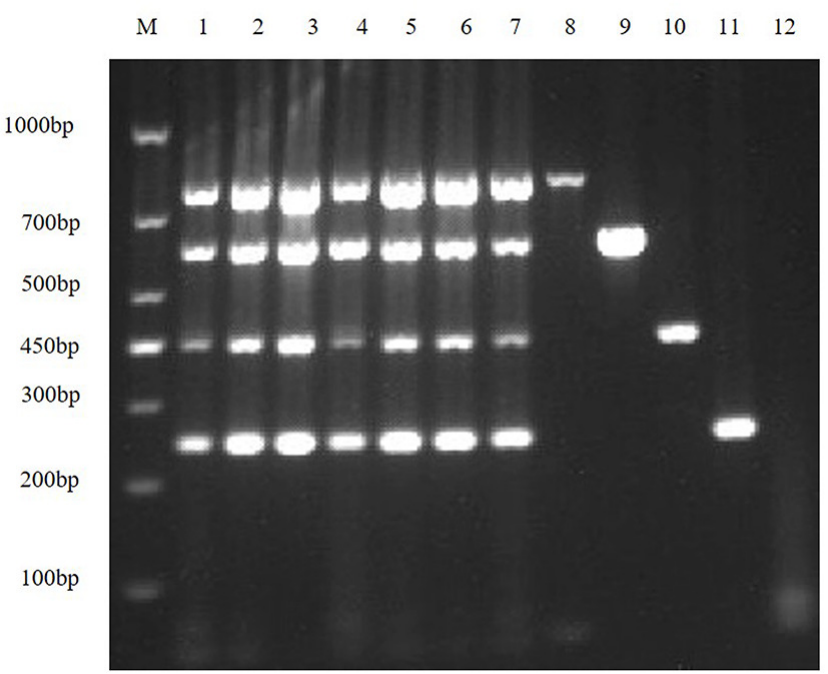
Optimize multiplex RT-PCR annealing temperature. This figure shows the optimization results of multiplex RT-PCR annealing temperature, lane M is DNA Marker DL1000, lanes 1–7 represent 7 temperature gradients of 53, 54, 55, 56, 57, 58, 59 °C; lanes 8–11 are the positive controls for TGEV, PEDV, SADS-CoV and PDCoV, respectively; lane 12 is the negative control.

First, the optimal annealing temperatures for monoplex RT-PCR primers for TGEV, PEDV, SADS-CoV and PDCoV were determined. Then, referring to the optimal annealing temperature of monoplex RT-PCR, and designing seven temperature gradients from 53 to 59°C. Finally, the optimal annealing temperature of multiplex RT-PCR is 55°C (Figure 2).

#### Optimizing the concentrations of TaKaRa Taq and DNTPs

**Figure 3:**
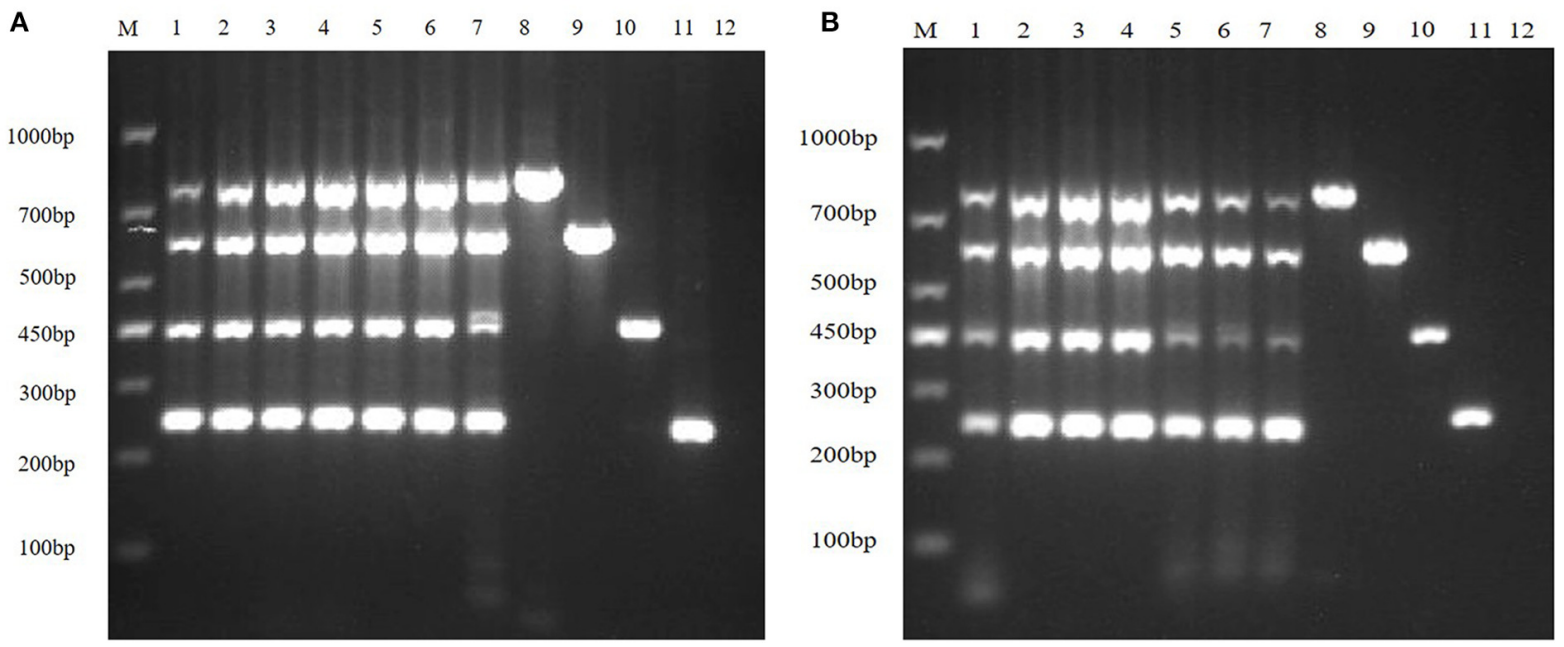
Optimizing the concentrations of TaKaRa Taq and dNTPs. **(A)** The optimized electrophoresis result of the optimal TaKaRa Taq concentration; lane M is DNA Marker DL1000, lanes 1–7 represent 0.02 U/μL, 0.04 U/μL, 0.06 U/μL, 0.08 U/μL, 0.10 U/μL, 0.12 U/μL, 0.16 U/μL; Lanes 8–11 are positive controls for TGEV, PEDV, SADS-CoV and PDCoV, respectively; and lane 12 is negative control. **(B)** The electrophoresis result of the optimal dNTPs concentration in multiplex RT-PCR; wherein, lane M is DNA Marker DL1000, and lanes 1–7 represent 0.10, 0.15, 0.20, 0.25, 0.30, 0.35, and 0.40 mM, respectively; lanes 8–11 are positive controls for TGEV, PEDV, SADS-CoV, and PDCoV, respectively; and lane 12 is a negative control.

In order to improve amplification efficiency, the optimal TaKaRa Taq concentration and the optimal dNTPs concentration were obtained by gradient RT-PCR amplification. The result of agarose gel electrophoresis showed that the optimal TaKaRa Taq concentration (Figure 3A) and the optimal dNTPs concentration (Figure 3B) for multiplex RT-PCR of TGEV, PEDV, SADS-CoV and PDCoV were 0.1 U/μL and 0.25 mM, respectively.

#### Multiplex RT-PCR primers optimization

**Figure 4:**
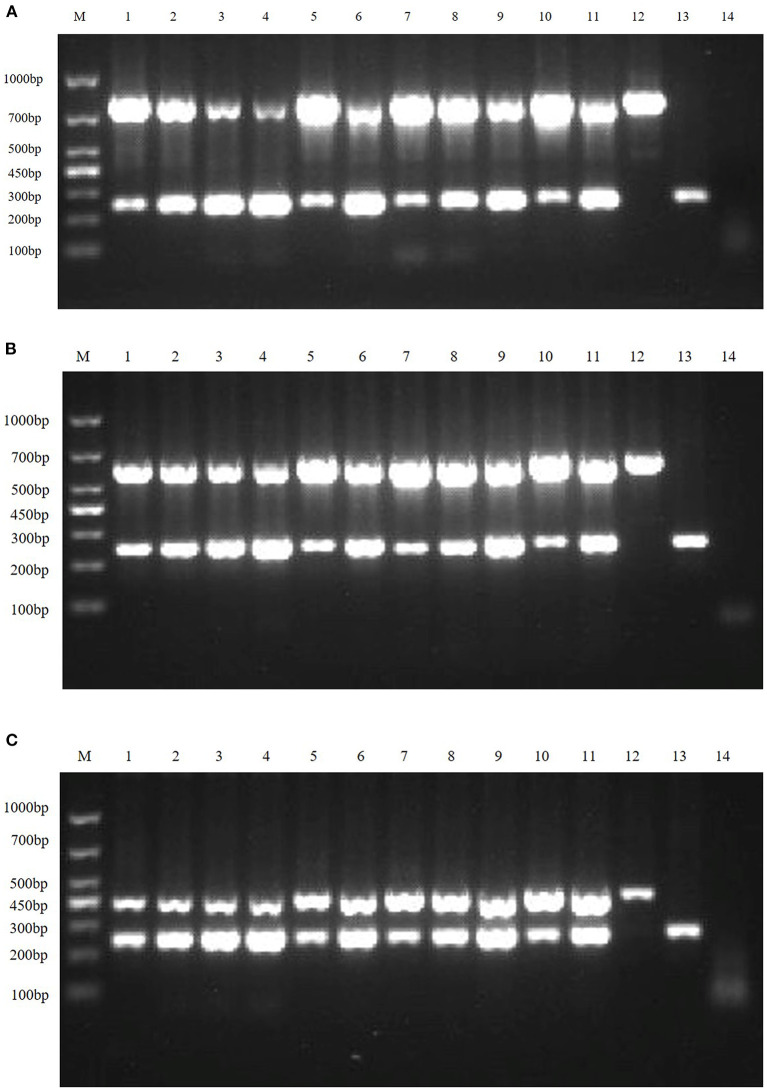
Multiplex RT-PCR primers optimization. **(A)** is the electrophoresis results of TGEV and PDCoV duplex RT-PCR with different primer ratios; lane M is DNA Marker DL1000; Lanes 1–11 are the primer ratios of 1: 1, 1: 2, 1: 3, 1: 4, 2: 1, 2: 3, 3: 1, 3: 2, 3: 4, 4: 1, 4: 3; Lanes 12–13 are the positive controls for TGEV and PDCoV; and lane 14 is the negative control. **(B)** The electrophoresis results of PEDV and PDCoV duplex RT-PCR with different primer ratios; lane M is DNA Marker DL1000; Lanes 1–11 are the primer ratios of 1: 1, 1: 2, 1: 3, 1: 4, 2: 1, 2: 3, 3: 1, 3: 2, 3: 4, 4: 1, 4: 3; Lanes 12–13 are the positive controls for PEDV and PDCoV; and lane 14 is the negative control. **(C)** The electrophoresis results of SADS-CoV and PDCoV duplex RT-PCR with different primer ratios; lane M is DNA Marker DL1000; Lanes 1–11 are the primer ratios of 1: 1, 1: 2, 1: 3, 1: 4, 2: 1, 2: 3, 3: 1, 3: 2, 3: 4, 4: 1, 4: 3; Lanes 12–13 are the positive controls for PEDV and PDCoV; and lane 14 is the negative control.

**Figure 5:**
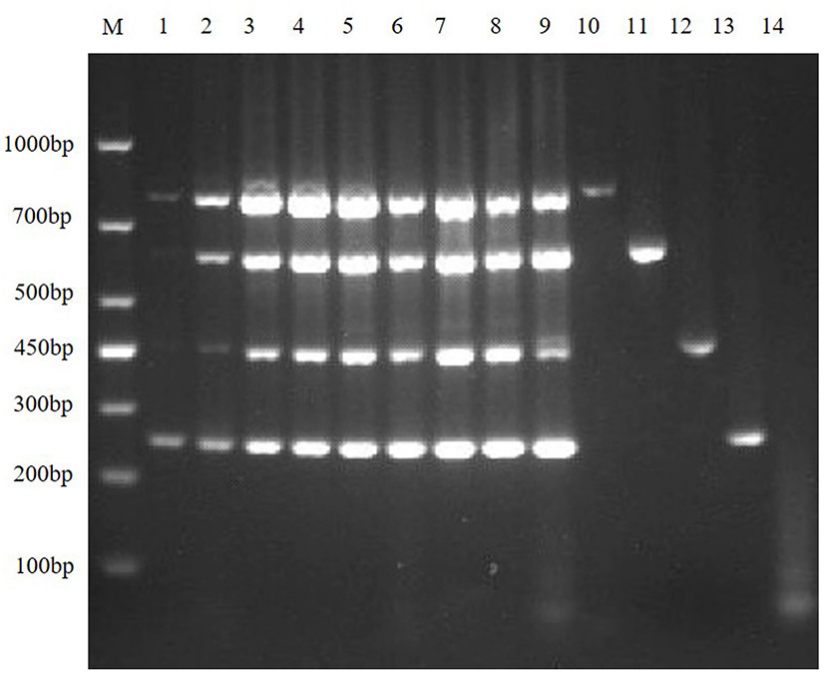
Primer concentration electrophoresis results of multiplex RT-PCR of TGEV, PEDV, SADS-CoV, and PDCoV. Lane M is DNA Marker DL1000, lanes 1–9 represent the concentration combination of nine primers in the fixed ratio (3:3:3:4) of TGEV, PEDV, SADS-CoV, and PDCoV; lanes 10–13 represent the positive control of TGEV, PEDV, SADS-CoV, and PDCoV; lane 14 was negative control.

Then, according to the analysis of the double RT-PCR optimization results, the final concentrations of the optimal primer combinations for each double RT-PCR are respectively: TGEV and PDCoV were 0.24 μM: 0.32 μM (3: 4) (Figure 4A); PEDV and PDCoV were 0.24 μM: 0.32 μM (3: 4) (Figure 4B); SADS-CoV and PDCoV were 0.24 μM: 0.32 μM (3:4) (Figure 4C, Table 3). Final primer concentrations were optimized by quadruplex RT-PCR reactions, ranging from 0.03 μM to 0.4 μM. The optimal final concentrations of primers were 0.24, 0.24, 0.24, 0.32 μM (3:3:3:4) (TGEV, PEDV, SADS-CoV, PDCoV) (Figure 5). Therefore, a primer concentration ratio of 3:3:3:4(TGEV, PEDV, SADS-CoV, PDCoV) was used as a standard to optimize primer concentrations for quadruplex PCR (Table 4).

**Table 3:** Ratio of duplex RT-PCR primers combination.

**Experimental group**	**PEDV** **(μM)**	**PDCoV** **(μM)**	**TGEV** **(μM)**	**PDCoV** **(μM)**	**SADS-CoV** **(μM)**	**PDCoV** **(μM)**	**Primer ratio**
1	0.20	0.20	0.20	0.20	0.20	0.20	1:1
2	0.20	0.40	0.20	0.40	0.20	0.40	1:2
3	0.20	0.60	0.20	0.60	0.20	0.60	1:3
4	0.20	0.80	0.20	0.80	0.20	0.80	1:4
5	0.40	0.20	0.40	0.20	0.40	0.20	2:1
6	0.40	0.60	0.40	0.60	0.40	0.60	2:3
7	0.60	0.20	0.60	0.20	0.60	0.20	3:1
8	0.60	0.40	0.60	0.40	0.60	0.40	3:2
9	0.60	0.80	0.60	0.80	0.60	0.80	3:4
10	0.80	0.20	0.80	0.20	0.80	0.20	4:1
11	0.80	0.20	0.80	0.20	0.80	0.20	4:3
12	0.20	0	0.20	0	0.20	0	$\text{P}{}_{1}^{+}$
13	0	0.20	0	0.20	0	0.20	P$\text{P}{}_{2}^{+}$
14	0.20	0.20	0.20	0.20	0.20	0.20	N

P (positive) represents positive, N (negative) represents negative; P^1+^ represents a positive control for PEDV, TGEV and SADS-CoV; P^2+^ represents the positive control for PDCoV, and N represents the negative control for the ratio of the two primer pairs.

**Table 4:** Primer concentration combinations at constant ratios.

**Experimental group**	**TGEV** **(μM)**	**PEDV** **(μM)**	**SADS-CoV** **(μM)**	**PDCoV** **(μM)**	**Primer ratio**
1	0.03	0.03	0.03	0.04	3:3:3:4
2	0.06	0.06	0.06	0.08	3:3:3:4
3	0.09	0.09	0.09	0.12	3:3:3:4
4	0.12	0.12	0.12	0.16	3:3:3:4
5	0.15	0.15	0.15	0.20	3:3:3:4
6	0.18	0.18	0.18	0.24	3:3:3:4
7	0.24	0.24	0.24	0.32	3:3:3:4
8	0.27	0.27	0.27	0.36	3:3:3:4
9	0.30	0.30	0.30	0.40	3:3:3:4

#### Sensitivity of the multiplex RT-PCR assay

**Figure 6:**
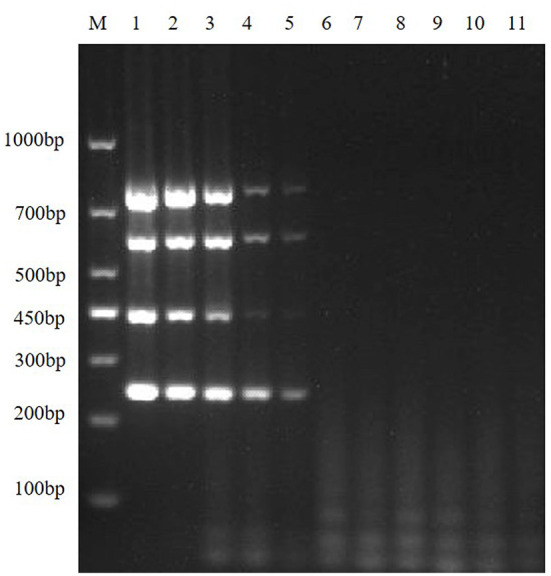
Sensitivity of the multiplex RT-PCR assay. This figure shows the results of sensitivity analysis of multiplex RT-PCR for TGEV, PEDV, SADS-CoV and PDCoV. Lane M is DNA Marker DL1000; lanes 1–10 represent the results of TGEV, PEDV, SADS-CoV, and PDCoV mixed plasmids diluted in a gradient of 10^0^-10^−9^, with a total of 10 template dilutions; lane 11 is a negative control.

In order to explore the LOD of the multiplex RT-PCR method, the positive recombinant plasmids of TGEV, PEDV, SADS-CoV and PDCoV were mixed, the standard plasmids were diluted in a gradient of 10^9^-10^0^, and use the diluted mixture as templates for RT-PCR amplification. The results showed that the sensitivities of multiplex RT-PCR to TGEV, PEDV, SADS-CoV and PDCoV were 8.54 × 10^5^ copies/μL, 6.48 × 10^5^ copies/μL, 7.79 × 10^6^ copies/μL and 5.66 × 10^5^ copies/μL, respectively (Figure 6).

#### Specificity of the multiplex RT-PCR assay

**Figure 7:**
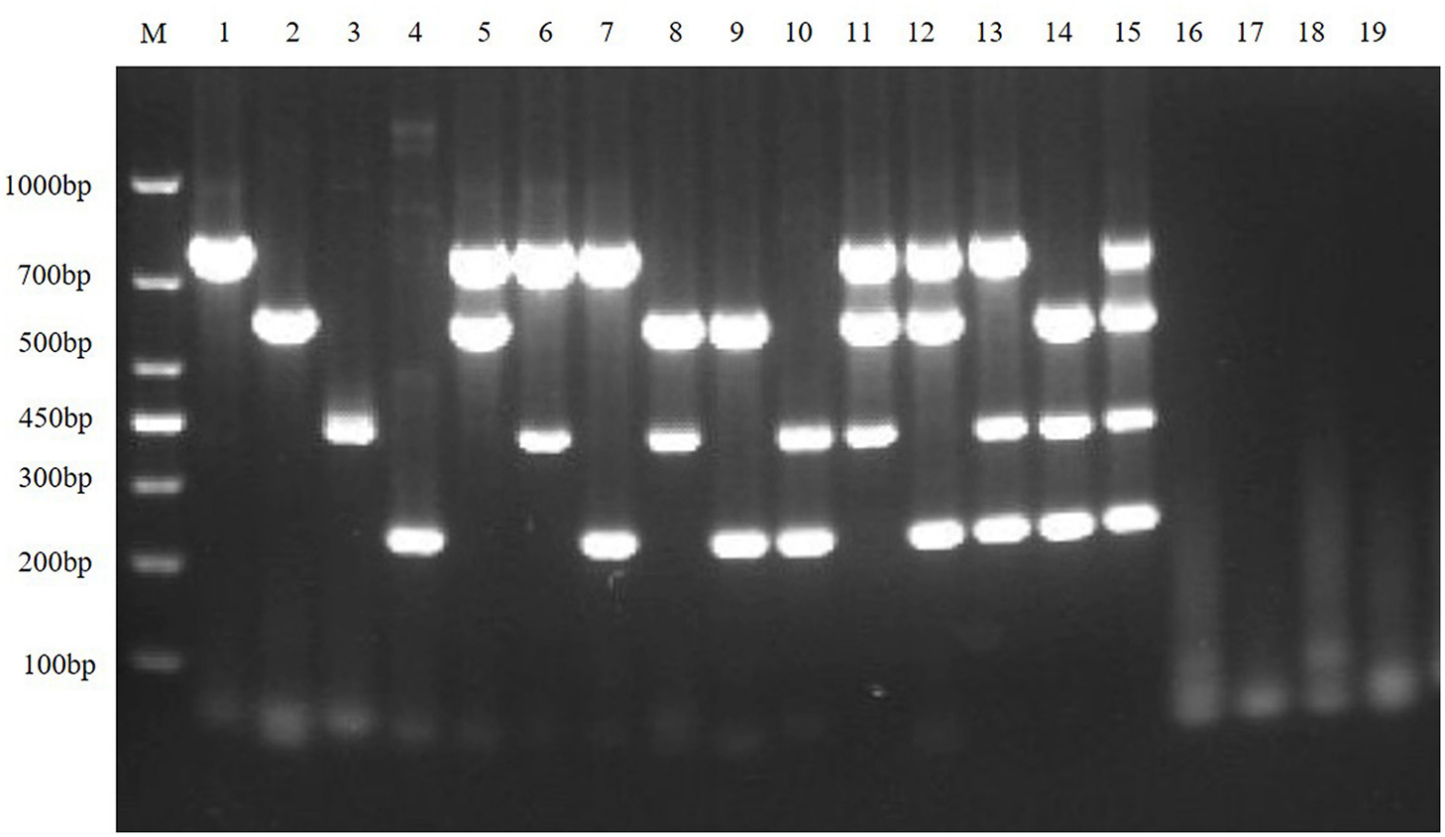
Specificity of the multiplex RT-PCR assay. This figure is the specificity analysis result of multiplex RT-PCR of TGEV, PEDV, SADS-CoV and PDCoV; lane M is DNA Marker DL1000, lanes 1–4 represent the monoplex RT-PCR specificity of TGEV, PEDV, SADS-CoV, and PDCoV, respectively; lanes 5–10 represent the duplex RT-PCR specificity of TGEV, PEDV, SADS-CoV and PDCoV, respectively; lanes 11–14 represent the triplex RT-PCR specificity of TGEV, PEDV, SADS-CoV, and PDCoV, respectively; lane 15 represents the quadruplex RT-PCR specificity of TGEV, PEDV, SADS-CoV, and PDCoV, respectively; lanes 16–19 represent SVA, PRRSV, and APPV, respectively; and lane 20 is the negative control.

To exclude potential false-positive results caused by other viruses that may be present in the sample, the quadruplex RT-PCR detection method was used to detect other virus-positive samples stored in our laboratory, including SVA, PRRSV, and APPV. The results showed that the multiplex RT-PCR method had well specificity (Figure 7).

#### Reproducibility test of the multiplex RT-PCR assay

**Figure 8:**
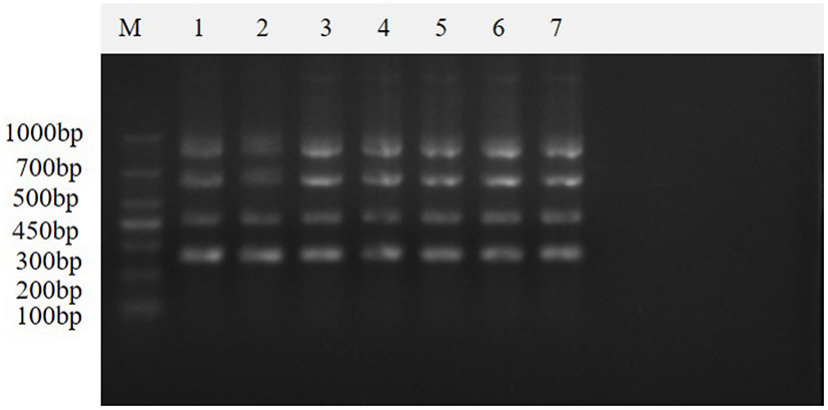
Repeatability of the multiplex real-time PCR assay. This figure shows the results of the repeatability assay of this method, which shows that the method is stable and reproducible. Lane M is DNA Marker DL1000; lanes 1–7 are the results of repeated experiments.

The results of seven repeated tests showed (Figure 8) that clear and uniform bands could be amplified in all seven tests, indicating that the method was stable and reproducible.

#### Clinical sample detection

**Figure 9:**
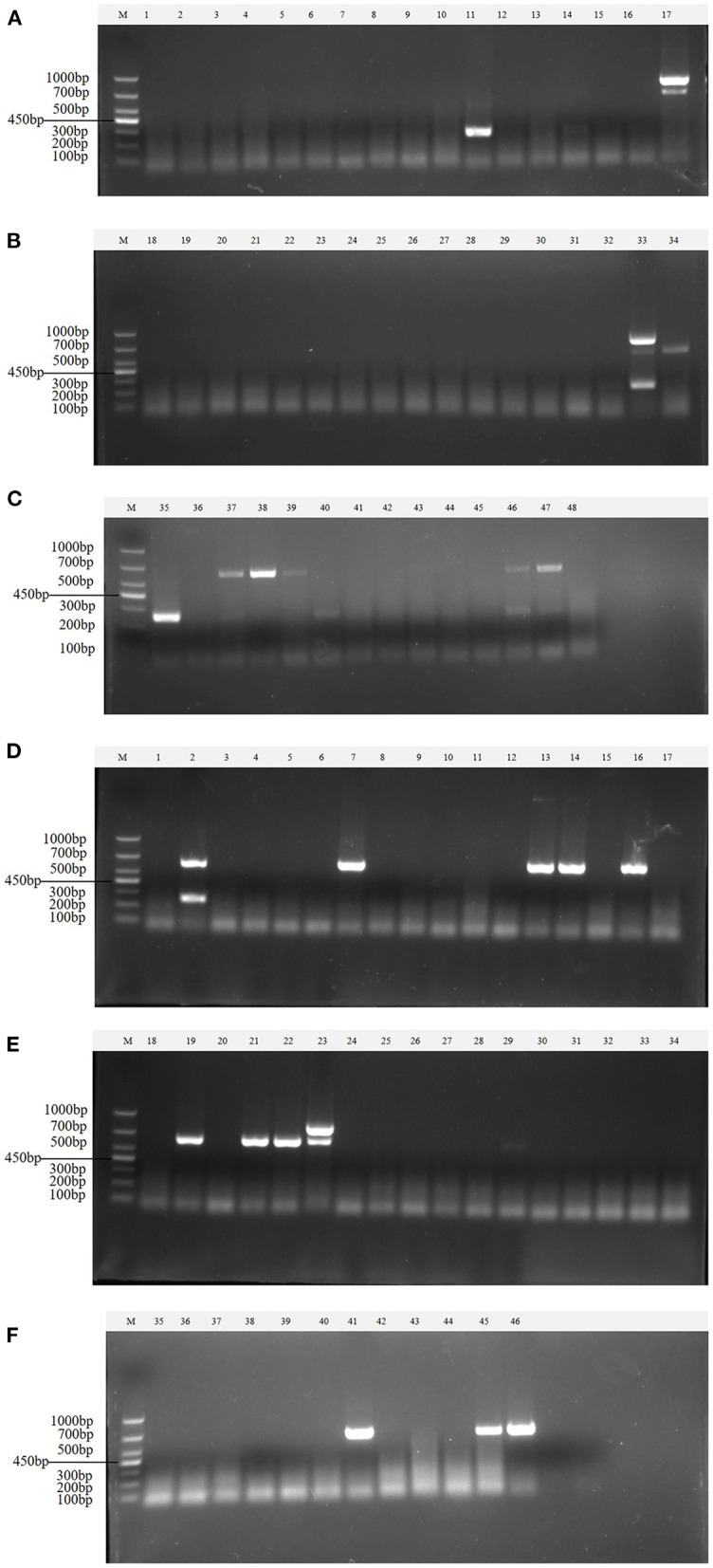
Detection in clinical samples. **(A–F)** The agarose gel electrophoresis pattern of the results of 94 clinical samples tested by multiplex RT-PCR. There were 15 positive samples for PEDV, 3 positive samples for mixed infection of PEDV and PDCoV, 2 positive samples for mixed infection of PEDV and TGEV, and 1 positive sample for mixed infection of PEDV, TGEV and PDCoV.

**Figure 10:**
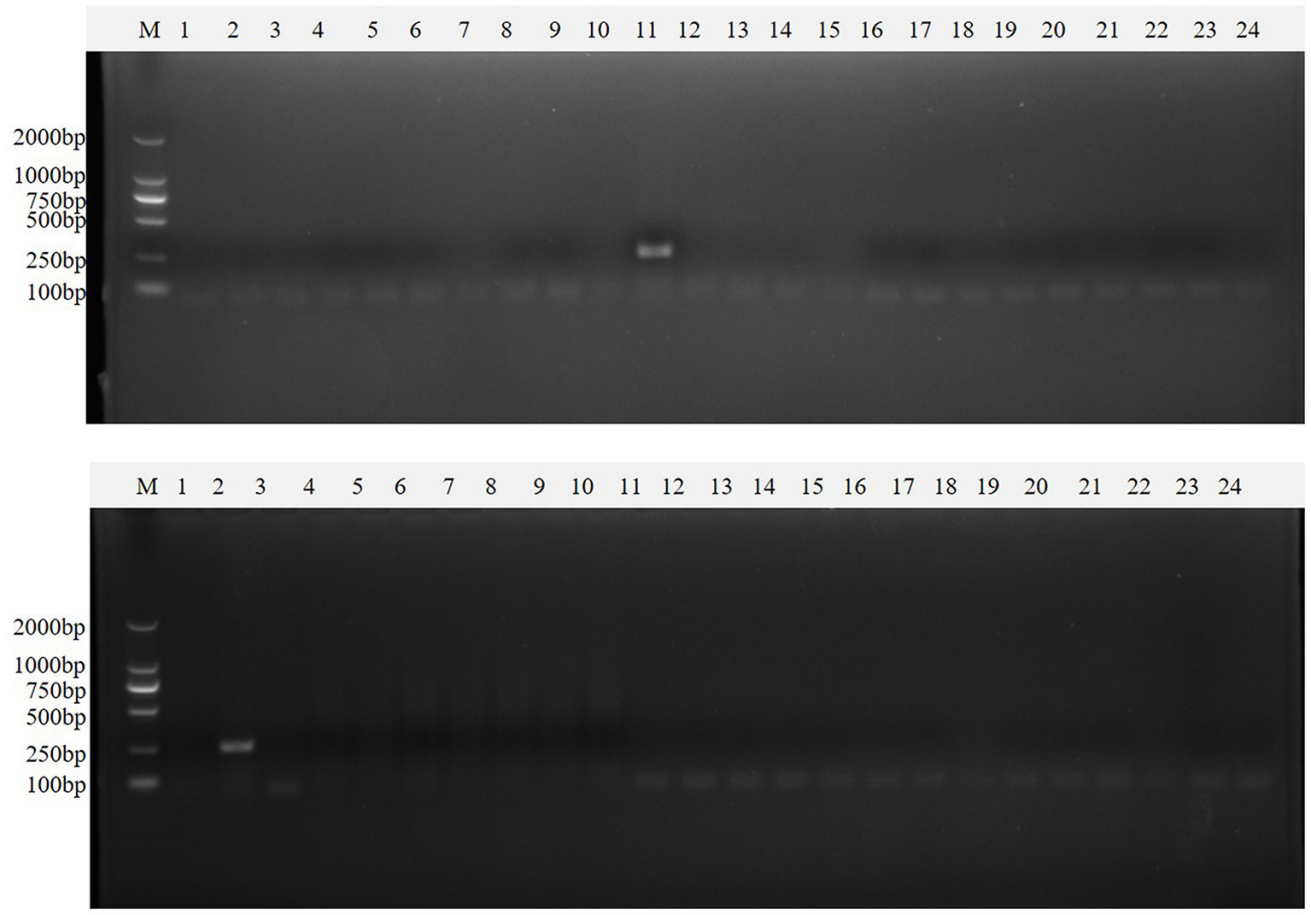
Single PT-PCR was used to verify the results of clinical samples. PDCoV **(A–C)** 1–24, PDCoV **(A–C)** 25–48, PDCoV **(D–F)** 1–24, and PDCoV **(D–F)** 25–46 represent agarose gel electrophoresis patterns of single RT-PCR recheck results of clinical samples tested for PDCoV; PEDV **(A–C)** 1–24, PEDV **(A–C)** 25–48, PEDV **(D–F)** 1–24 and PEDV **(D–F)** 25–46 represent agarose gel electrophoresis patterns of single RT-PCR recheck results of clinical samples tested for PEDV; SADS-CoV **(A–C)** 1–24, SADS-CoV **(A–C)** 25–48, SADS-CoV **(D–F)** 1–24 and SADS-CoV **(D–F)** 25–46 represent agarose gel electrophoresis patterns of single RT-PCR recheck results of clinical samples tested for SADS-CoV; TGEV **(A–C)** 1–24, TGEV **(A–C)** 25–48, TGEV **(D–F)** 1–24 and TGEV **(D–F)** 25–46 represent agarose gel electrophoresis patterns of single RT-PCR recheck results of clinical samples tested for TGEV.

Clinical samples were evaluated using the method established in this study, 94 samples collected from pig farms to validate their performance in clinical applications. After identification, there were 15 positive samples for PEDV, three positive samples for mixed infection of PEDV and PDCoV, 2 positive samples for mixed infection of PEDV and TGEV, and 1 positive sample for mixed infection of PEDV, TGEV and PDCoV (Figure 9). The coincidence rate of the multiplex RT-PCR assay and the conventional single RT-PCR assay in the detection of clinical samples was 100% (Table 5, Figure 10).

**Table 5:** Results of clinical samples detected by the multiplex RT-PCR.

**Pathogens**	**Singleplex RT-PCR**			**Multiplex RT-PCR**			**Coincidence rate (%)**
	**Sample number**	**Positive**	**Percentage (%)**	**Sample number**	**Positive**	**Percentage (%)**	
PEDV	94	15	15.96	94	15	15.96	100
TGEV	94	0	0	94	0	0	100
PDCoV	94	4	4.26	94	4	4.26	100
SADS-CoV	94	0	0	94	0	0	100
PEDV+TGEV	94	2	2.13	94	2	2.13	100
PEDV+PDCoV	94	3	3.19	94	3	3.19	100
PEDV+PDCoV+TGEV	94	1	1.06	94	1	1.06	100

## Discussion

Porcine viral diarrhea is widespread in the world and spreads rapidly, causing huge economic losses to the global swine industry. These enteroviruses can cause vomiting, diarrhea, and dehydration in infected pigs. In severe cases, a large number of piglets died and the damage was very serious (22). These enteroviruses have similar characteristics after infecting pigs and require laboratory testing to differentiate them. In addition, there are often mixed infections in clinical cases, which brings challenges to the prevention, control and treatment (2). Therefore, establishing a detection method for simultaneous detection of multiple pathogens will greatly improve the diagnosis and prevention and control of swine diarrheal diseases.

Diarrheal virus infection causes diarrhea, high mortality in piglets. Common porcine enteric coronaviruses include TGEV, PEDV, PDCoV, and the SADS-CoV (23, 24) could seriously endanger the development of the pig industry, especially in terms of newborn piglets. The symptoms caused by the above mentioned four viruses are similar, so it is difficult to determine the causative pathogen in clinical diagnosis. Moreover, relatively few studies examine newly-epidemic diseases. Therefore, a rapid, specific, and low-cost detection method is sorely needed for the surveillance of diarrhea viruses.

In recent years, traditional monoplex RT-PCR methods, multiplex RT-PCR methods and multiplex RT-qPCR methods targeting conserved regions have been established for some viruses (21, 24, 25). RT-qPCR-based methods have the disadvantages of high cost and high instrument requirements, and many laboratories cannot obtain relatively expensive qPCR machines. Traditional monoplex RT-PCR requires multiple tests to determine the final result, and the process is cumbersome. The multiplex RT-PCR adds multiple pairs of primers to the same RT-PCR reaction system to detect multiple target genes at the same time, which improves the detection efficiency, and has the sensitivity and specificity of a single RT-PCR; compared with qPCR, it is extensively used, and inexpensive. Therefore, we developed multiplex RT-PCR to detect and differentially diagnose four diarrheal viruses in swine herds. In this study, a multiplex RT-PCR assay was established. For the best amplification efficiency, the final concentrations of multiple pairs of primers, TaKaRa Taq enzyme and dNTP Mixture in the reaction system were optimized, and the annealing temperature of the reaction program was optimized (21). Compared with traditional monoplex PCR primers, four viruses can be detected in a single reaction. The specificity of this method showed that each pair of primers could only detect the target gene itself, but could not detect the non-specific fragment. The sensitivity results showed that the samples with the mixture of four kinds of positive plasmid still had excellent sensitivity.

According to the virus detection and source analysis of positive samples in this study, the results show that PEDV, TGEV, and PDCoV are still the main causes of pig diarrhea in South China, which is consistent with previous research results (26). Porcine viral diarrheal disease may be caused by a single virus or a combination of multiple viruses, and coinfection of porcine enteroviruses has been reported to be common in pig farm. The results of these clinical samples confirmed the cases of mixed infection of the viruses in pigs. During the epidemiological investigation, our laboratory found that among mixed infections, the co-infection rate of PEDV and PDCoV was the highest at 9.39%, followed by PEDV and SADS-CoV at 7.18% (24). Epidemiological surveys showed that SADS-CoV was only found in Guangdong and Fujian provinces, and not in other areas. Therefore, it is necessary to strengthen the surveillance of SADS-CoV to prevent its spread to other areas. The latest research shows that coronaviruses can spread across species. According to research, mixed infection may lead to recombination between viruses and changes in virus virulence. This highlights the importance of identifying multiple viral infections simultaneously.

In conclusion, the established multiplex RT-PCR method has excellent specificity, well detection efficiency and can be applied to laboratory diagnosis, epidemiological research and monitoring of SADS-CoV, TGEV, PEDV, and PDCoV. In addition, the established method can be applied to the clinical differential diagnosis of clinical mixed infection, and realize the early diagnosis of clinical cases.

## Data availability statement

The original contributions presented in the study are included in the article/supplementary material, further inquiries can be directed to the corresponding author.

## Author contributions

D-SH, S-LZ, and R-AC were involved in the experimental design and provided guidance on the experimental operation. J-WN, J-HL, J-LG, K-HD, X-WW, GL, XZ, and M-SX performed the experiments and data analysis. All authors contributed to writing the manuscript and have read and approved the final version of the manuscript.

## Funding

This study was supported by the following grants: Key Laboratory of Zoonosis Prevention and Control of Guangdong Province (No. P20211154-0302), the Guangdong Province Pig Industrial System Innovation Team (No. 2018LM1103), Key-Area R&D Program of GuangDong Province (No. 2021B0707010009), Major Program of Zhaoqing Branch Center of Guangdong Laboratory for Lingnan Modern Agricultural Science and Technology (No. P20211154-0101), Guangdong Department of Science and Technology (No. 2020B0202080004), Guangzhou Science and Technology Bureau (Nos. 202103000096 and 202206010192), Guangdong Provincial Science and Technology Planning Project (No. P20211154-030), and the grant (No. 2021TDQD002) from Maoming Branch Center of Guangdong Laboratory for Lingnan Modern Agricultural Science and Technology.

## Conflict of interest

The authors declare that the research was conducted in the absence of any commercial or financial relationships that could be construed as a potential conflict of interest.

## Publisher's note

All claims expressed in this article are solely those of the authors and do not necessarily represent those of their affiliated organizations, or those of the publisher, the editors and the reviewers. Any product that may be evaluated in this article, or claim that may be made by its manufacturer, is not guaranteed or endorsed by the publisher.
